# Context-dependent role for chromatin remodeling component PBRM1/BAF180 in clear cell renal cell carcinoma

**DOI:** 10.1038/oncsis.2016.89

**Published:** 2017-01-16

**Authors:** A Murakami, L Wang, S Kalhorn, P Schraml, W K Rathmell, A C Tan, R Nemenoff, K Stenmark, B-H Jiang, M E Reyland, L Heasley, C-J Hu

**Affiliations:** 1 Molecular Biology Graduate Program, University of Colorado Anschutz Medical Campus, Aurora, CO, USA; 2 Department of Craniofacial Biology, School of Dental Medicine, University of Colorado Anschutz Medical Campus, Aurora, CO, USA; 3 Doctor of Dental Surgery Program, School of Dental Medicine, University of Colorado Anschutz Medical Campus, Aurora, CO, USA; 4 Institute of Surgical Pathology, University Hospital Zurich, Zurich, Switzerland; 5 Division of Hematology/Oncology, Vanderbilt-Ingram Cancer Center, Vanderbilt University Medical Center, Nashville, TN, USA; 6 Division of Medical Oncology, Department of Medicine, University of Colorado Anschutz Medical Campus, Aurora, CO, USA; 7 Division of Renal and Hypertension, Department of Medicine, University of Colorado Anschutz Medical Campus, Aurora, CO, USA; 8 Departments of Pediatrics, Medicine, and Anesthesiology, School of Medicine, University of Colorado Anschutz Medical Campus, Aurora, CO, USA; 9 Department of Pathology, Anatomy and Cell Biology, Thomas Jefferson University, Philadelphia, PA, USA

## Abstract

A subset of clear cell renal cell carcinoma (ccRCC) tumors exhibit a *HIF1A* gene mutation, yielding two ccRCC tumor types, H1H2 type expressing both HIF1α and HIF2α, and H2 type expressing HIF2α, but not functional HIF1α protein. However, it is unclear how the H1H2 type ccRCC tumors escape HIF1's tumor-suppressive activity. The polybromo-1 (*PBRM1*) gene coding for the BAF180 protein, a component of the SWItch/Sucrose Non-Fermentable (SWI/SNF) chromatin remodeling complex, is inactivated in 40% ccRCCs, the function and mechanism of BAF180 mutation is unknown. Our previous study indicates that BAF180-containing SWI/SNF chromatin remodeling complex is a co-activator for transcription factor HIF to induce HIF target genes. Thus, our questions are if BAF180 is involved in HIF-mediated hypoxia response and if *PBRM1/BAF180* mutation has any association with the HIF1A retention in H1H2 type ccRCC. We report here that BAF180 is mutated in H1H2 ccRCC cell lines and tumors, and BAF180 re-expression in H1H2 ccRCC cell lines reduced cell proliferation/survival, indicating that BAF180 has tumor-suppressive role in these cells. However, BAF180 is expressed in HIF1-deficient H2 ccRCC cell lines and tumors, and BAF180 knockdown in H2 type ccRCC cell lines reduced cell proliferation/survival, indicating that BAF180 has tumor-promoting activity in these cells. In addition, our data show that BAF180 functions as co-activator for HIF1- and HIF2-mediated transcriptional response, and BAF180's tumor-suppressive and -promoting activity in ccRCC cell lines depends on co-expression of HIF1 and HIF2, respectively. Thus, our studies reveal that BAF180 function in ccRCC is context dependent, and that mutation of *PBRM1/BAF180* serves as an alternative strategy for ccRCC tumors to reduce HIF1 tumor-suppressive activity in H1H2 ccRCC tumors. Our studies define distinct functional subgroups of ccRCCs based on expression of BAF180, and suggest that BAF180 inhibition may be a novel therapeutic target for patients with H2, but not H1H2, ccRCC tumors.

## Introduction

The incidence of kidney cancers has continued to rise, with 62 000 new cases and over 14 000 deaths predicted to occur in 2016 in the United States.^1^ Kidney cancer is one of the genitourinary tract cancers that have high mortality rate^2, 3, 4, 5, 6^ due to a paucity of effective treatments, indicating an urgent need to better understand the biology of kidney cancer.

The majority of kidney cancers are clear cell renal cell carcinomas (ccRCC).^2, 4^ Recent exome sequencing of ccRCC tumors has identified almost universal mutation of the von-Hippel Lindau (*VHL*) tumor-suppressive gene.^7, 8, 9, 10, 11^ pVHL is a component of the E3 ubiquitin ligase complex, and regulates stability of the transcription factors (TFs), HIF1α and HIF2α.^12^ In cells with wild-type pVHL, HIF1α and HIF2α proteins are targeted for proteasome degradation under normoxic conditions, but stabilized when cells become hypoxic (HX). However, in the absence of a functional pVHL protein, HIF1α and HIF2α are stabilized even under normoxic conditions. HIF1α and HIF2α dimerize with the Aryl Hydrocarbon Receptor Nuclear Translocator (ARNT) to form HIF1α/ARNT (HIF1) or HIF2α/ARNT (HIF2) complexes that induce expression of the genes that regulate metabolism, angiogenesis, cell proliferation and the epithelial-to-mesenchymal transition.^13, 14, 15^ Thus, HIF-mediated transcription response is critically important for ccRCC disease.

Despite the significant structural similarities of HIF1α and HIF2α proteins, and their common co-factor, ARNT, HIF1 and HIF2 regulate unique, as well as common target genes.^16, 17^ Thus, it is not surprising that both *HIF1A* and *HIF2A* gene are required for the ccRCC tumor development/initiation in a mouse model, in which both *VHL* and *TP53* are specifically knocked out in renal tubule epithelial cells.^18^ Despite positive role of both HIF1 and HIF2 in ccRCC initiation, results from clinical and laboratory studies indicate that HIF2 plays a positive role in ccRCC tumor maintenance,^19, 20, 21^ whereas HIF1 has a tumor-suppressive role in late stage ccRCC development and in established ccRCC tumors. On the basis of the expression pattern of HIF1α, ccRCC tumors can be divided into two subtypes: H2 ccRCC tumors that express HIF2α but not a functional HIF1α protein, and H1H2 ccRCC tumors that express both HIF1α and HIF2α protein.^2, 22^ Given the evidence that HIF1 functions as a tumor suppressor, an important question that has not been addressed is how H1H2 ccRCC tumors tolerate HIF1α protein expression.

Exome sequencing has revealed that 40% of ccRCC tumors also harbor mutations in the polybromo-1 (*PBRM1*) gene that codes for the BAF180 protein (we will use the term of BAF180 for the gene and the protein, for simplicity), an accessory component of the PBAF complex, one type of SWI/SNF chromatin remodeling complex.^7, 8, 9, 10, 11^ SWI/SNF chromatin remodeling complexes regulate gene transcription through nucleosome disruption and reconstruction in an ATP-dependent manner. Our laboratory has previously demonstrated that the BAF180-containing SWI/SNF chromatin remodeling complex, PBAF complex, acts as a co-activator for HIF1- and HIF2-mediated transcriptional responses by remodeling the promoters of HIF target genes.^23^ These data support a hypothesis that the *PBRM1* gene product, BAF180, a unique component of the PBAF complex, may also be important for the HIF-mediated hypoxia response and *PBRM1/BAF180* gene mutation may reduce the tumor-suppressive activity of HIF1 in H1H2 ccRCCs. Thus, the goal of this study was to determine if PBRM1/BAF180 is important for HIF1- and HIF2-mediated transcriptional response, and if the *BAF180* gene mutation is associated with *HIF1A* retention in H1H2 ccRCC, a tumor-suppressive factor in established ccRCC tumors. Elucidating the function and molecular mechanism of *BAF180* mutation may provide novel therapeutic target for ccRCC patients.

## Results

### Mutually exclusive expression of BAF180 and HIF1α protein in ccRCC cell lines

**Figure 1:**
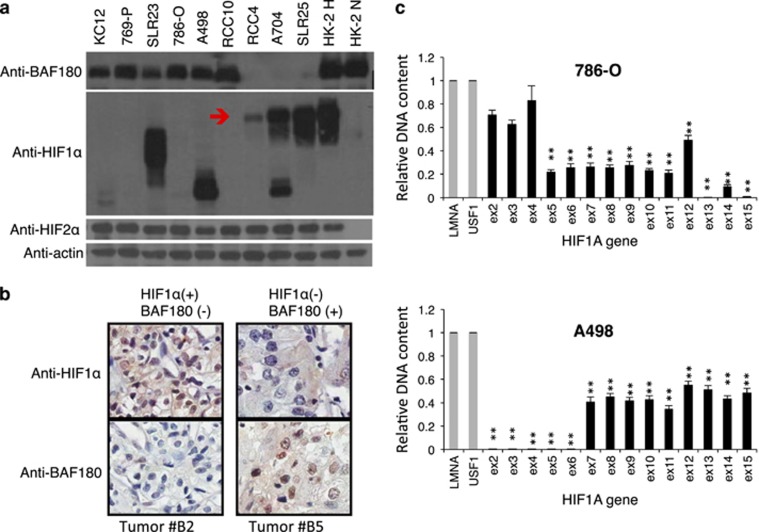
Mutually exclusive expression of full-length BAF180 and HIF1α proteins in ccRCC cell lines and some primary tumors. (**a**) Western blot analysis of BAF180, HIF1α and HIF2α protein in ccRCC cell lines. The red arrow indicates the position of the full-length HIF1α protein. A704 cells express full-length as well as truncated HIF1α protein. Normoxic and hypoxic HK2 cells were used as positive controls for BAF180, HIF1α and HIF2α protein detection. (**b**) Immunohistochemistry staining of HIF1α or BAF180 proteins in the #B2 and #B5 ccRCC tumors in the ccRCC tumor microarray (US Biomax, Derwood, MD, USA, cat. #BC07014a). (**c**) qPCR analysis of the abundance of individual exons of the *HIF1A* gene in genomic DNAs isolated from the indicated ccRCC cell lines. Relative DNA content was normalized to *LMNA* and *USF1* genes as these genes were not amplified or deleted in ccRCC cells. In addition, DNA content of *HIF1A* exons from HK2 cells were used as calibrators as *HIF1A* gene is maintained at two copies in HK2 cells. Exons with relative DNA content at 1, 0.5 or close to zero indicate normal, loss of one copy or loss of both alleles in ccRCC cell lines.

HIF1 exhibits tumor-suppressive effects in established ccRCC tumors,^24, 25^ but is still expressed in a subset of ccRCC tumors. Further, the BAF180-containing SWI–SNF chromatin remodeling complex is critical for HIF1-mediated transcriptional response and BAF180 is mutated in a subset of ccRCC tumors.^7, 8, 9, 10, 11, 23^ These observations prompted us to test the hypothesis that *BAF180* mutation is associated with *HIF1A* retention in H1H2 ccRCC tumors. Thus, we assessed BAF180, HIF1α and HIF2α protein expression across a panel of ccRCC cell lines (Figure 1a). Consistent with its oncogenic role, HIF2α protein was detected in all ccRCC cell lines under normoxia (Figure 1a). However, HIF1α is lost in KC-12, 769-P, 786-O, RCC10 or truncated in SLR 23 and A498 cells, whereas BAF180 protein expression is lost in RCC4, A704 and SLR25 cell lines (Figure 1a). Interestingly, there is a relationship between BAF180 and HIF1α protein expression, in which cell lines lack BAF180 protein expression (SLR25, A704 and RCC4) expressed full-length HIF1α protein (Figure 1a, indicated by a red arrow), whereas the BAF180-expressing ccRCC cell lines lacked HIF1α protein detection (KC-12, 769-P, 786-O and RCC10) or expressed truncated/non-functional HIF1α proteins (SLR23 and A498; Figure 1a). We next examined HIF1α and BAF180 protein expression in a ccRCC tumor tissue microarray. Twenty-two out of a total of 66 ccRCC tumors exhibited a HIF1α^+^/BAF180^−^ pattern, 7 tumors were HIF1α^−^/BAF180^+^, 36 were double positive and 3 were double negative. Representative images from HIF1α^+^/BAF180^−^ and HIF1α^−^/BAF180^+^ ccRCC tumors are shown (Figure 1b). Thus, expression of full-length HIF1α and BAF180 protein may have a mutually exclusive relationship in most ccRCC cell lines and in some primary tumors.

### *HIF1A* gene mutation is mainly by exon deletion in ccRCC cell lines

To determine the molecular mechanism of loss of HIF1α protein expression, or expression of truncated HIF1α protein in ccRCC cell lines, we quantified the abundance of individual exons of the *HIF1A* gene in genomic DNAs isolated from ccRCC cell lines (Figure 1c and Supplementary Figure 1). Using the non-altered genes of lamin A/C (*LMNA*) and upstream stimulatory factor 1 (*USF1*) as DNA-loading controls, and genomic DNA from normal kidney epithelial HK2 cells as a normal DNA copy number control, most HIF1α-defective ccRCC cell lines were found to have loss of one *HIF1A* allele and loss of several exons of the second allele of the *HIF1A* gene (Figure 1c and Supplementary Figure 1). For example, 786-O cells lost exons 5–15 in one *HIF1A* allele and had a deletion of exons 13–15 in the second allele, also A498 cells lost one *HIF1A* allele completely and had additional deletion of exons 2–6 in the second allele. Our data are consistent with previous reports^10, 11, 26, 27^ that 14q33.1 where the *HIF1A* gene is located, is often associated with copy number loss in ccRCC tumors.

### BAF180 has distinct functions in H1H2 and H2 ccRCC cell lines

**Figure 2:**
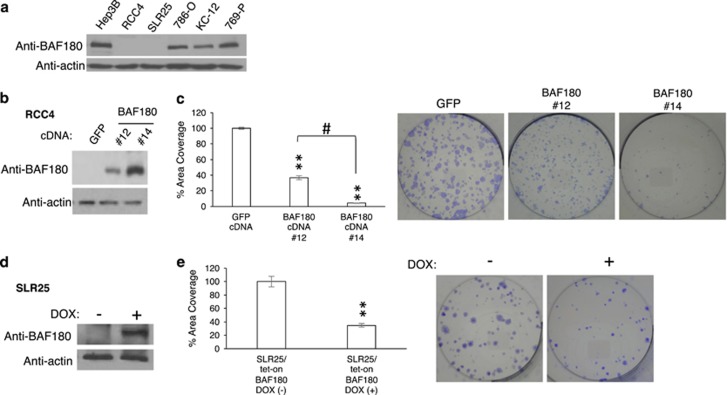
Re-expression of BAF180 in H1H2 ccRCC lines of RCC4 and SLR25 reduces cell survival/proliferation. (**a**) Western blot analysis of BAF180 protein in ccRCC cell lines. Beta actin serves as a protein-loading control for this and other WB analysis in the paper. (**b**) Western blot analysis of BAF180 protein in RCC4 cell clones stably transfected with a *BAF180* expression vector or a vector-expressing *GFP*. (**c**) Quantification and photos of clonogenic survival assays for RCC4/BAF180 or GFP cells. Quantification was performed using MetaMorph software to measure the total area covered by colonies. (**d**) Western blot analysis of BAF180 protein expression in SLR25/Tet-on BAF180 cell line with (+) or without (−) doxycycline treatment. (**e**) Quantification and photos of clonogenic survival assay for SLR25/Tet-on BAF180 cells in the presence (+) or absence (−) of doxcycline.

To determine the effect of BAF180 protein re-expression in BAF180-deficient ccRCC cell lines, individual RCC4 clones with varying amounts of BAF180 protein re-expression were generated (Figure 2b). A clonogenic assay determined that re-expression of BAF180 decreased the cell survival/proliferation of RCC4 cells in a dose-dependent manner (Figure 2c). Likewise, BAF180 re-expression in a doxycycline-inducible manner also reduced SLR25 cell survival/proliferation (Figures 2d and e). Taken together, these results demonstrated that BAF180 re-expression reduces cell survival/proliferation in BAF180-deficient RCC4 and SLR25 cells, consistent with a reported tumor-suppressive role of BAF180 in some ccRCC cell lines.^7, 28, 29^

**Figure 3:**
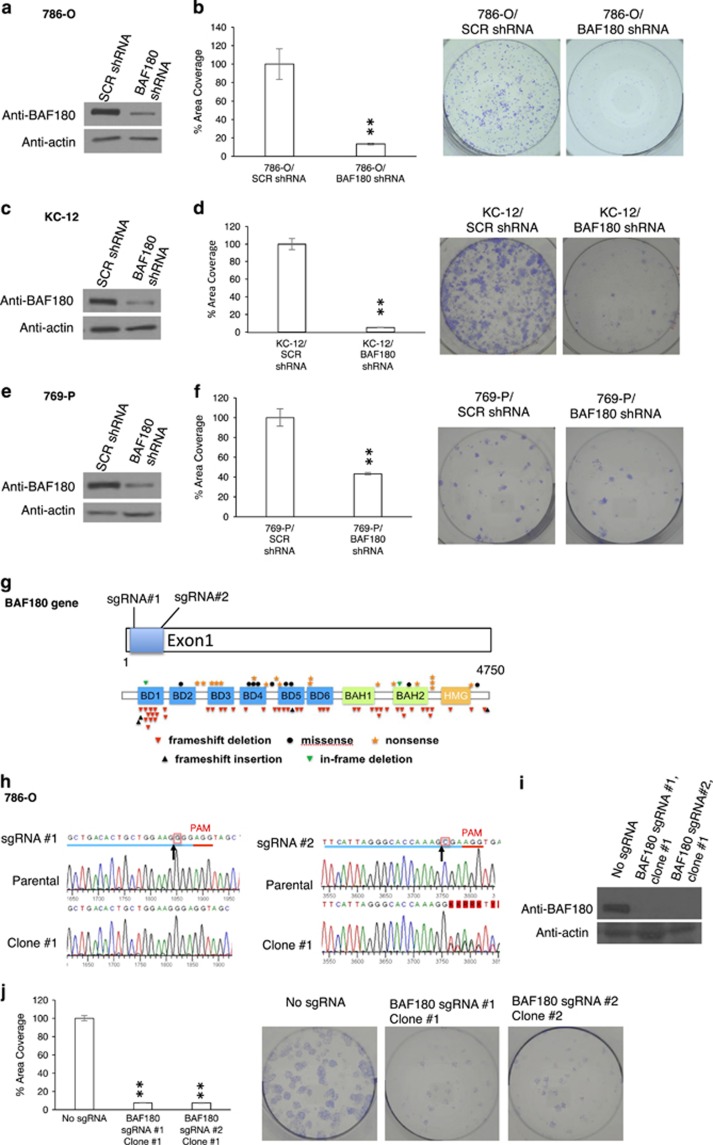
BAF180 knockdown in BAF180-expressing H2 ccRCC cell lines reduces cell survival/proliferation. (**a**, **c**, **e**) Western blot analyses of BAF180 protein in 786-O (**a**), KC-12 (**c**) and 769-P (**e**) H2 ccRCC cells targeted with non-targeting scramble (SCR) shRNA or BAF180 shRNA. (**b**, **d**, **f**) Quantification and photos of the clonogenic survival assay for 786-O (**b**), KC-12 (**d**) and 769-P (**f**) cells targeted with control scr-shRNA or BAF180 shRNA. (**g**) The location of BAF180 sgRNA #1 and #2 relative to the BAF180-coding region are shown (upper). The diagram (lower) shows the type and frequency of mutations in the *BAF180* gene, found in ccRCC tumors, adapted from the article by Valera *et al.*^7^ (**h**) DNA sequence and sequencing profiles of parental *BAF180* DNA (parental) and *BAF180* DNA after targeted by BAF180 sgRNA #1 (left) and #2 (right). The blue lines indicate the target sequences of sgRNAs and the red lines showing the protospacer adjacent motif sequence. The black arrows indicate the positions that double-strand DNA cleavages are expected to occur by the sgRNA-led Cas9 enzyme. The red boxes are to indicate the nucleotides that have been deleted by CRISPR–Cas9 system. SgRNA #2 Clone #1 shows a mixed sequence after the cleavage site, probably due to the fact that the deletion in the *BAF180* allele 1 is different from the deletion in *BAF180* allele 2. (**i**) Western blot analysis of BAF180 protein in 786-O/Cas9 cells expressing no sgRNA, or PBRM1 sgRNA #1 (clone #1) or #2 (clone #1). (**j**) Quantification and photos of the clonogenic survival assay for 786-O/Tet-on Cas9 clones without sgRNA expression, or with expression of BAF180 sgRNA #1 (clone #1) or #2 (clone #1).

To determine if BAF180 also exhibits tumor-suppressive activity in BAF180-expressing ccRCC cell lines (Figure 2a; 786-O, KC-12 and 769-P), we first decreased BAF180 protein levels in 786-O cells using *BAF180* short hairpin RNA (shRNA; Figure 3a). Surprisingly, clonogenic assays revealed that 786-O/*BAF180* shRNA cells exhibited a significant reduction in their clonogenic survival/proliferation (Figure 3b). *BAF180* knockdown in KC-12 and 769-P cells (Figures 3d and f), as well as RCC10 and A498 (not shown) also decreased cell survival/proliferation.

To definitively determine the function of BAF180 in BAF180-expressing ccRCC cell lines, we knocked out *BAF180* gene expression in 786-O cells using CRISPR–Cas9 technology. Expression of a *BAF180* single-guide RNA #1 (sgRNA#1) and Cas9 nuclease are expected to introduce double-stranded DNA breaks and subsequently mutation in exon 1 of the *BAF180* gene, a location where *BAF180* mutations are frequently observed in primary ccRCC tumors^7, 10, 11^ (Figure 3g). All three CRISPR/Cas9 BAF180-deficient clones were determined to contain one nucleotide deletion, after the expected cleavage site (Figure 3h; sgRNA #1, clone #1 and not shown). This mutation led to a premature stop codon within 30 amino acids after the deleted nucleotide and complete loss of BAF180 protein expression (Figure 3i and not shown). A clonogenic assay showed that 786-O/*BAF180* sgRNA #1 clones #1, #2 and #3 exhibited significantly reduced cell survival/proliferation (Figure 3j and not shown). To control for potential off-target effects of *BAF180* sgRNA #1, we used another *BAF180* sgRNA (sgRNA #2 in Figure 3g), also located at BAF180 exon 1. Similarly, *BAF180* sgRNA #2 led to the nucleotide deletion (Figure 3h and not shown), which resulted in a loss of BAF180 protein expression (Figure 3i and not shown) and a reduced cell survival/proliferation (Figure 3j and not shown). Taken together, these data indicate that BAF180 protein has a tumor-suppressive role in BAF180-deficient ccRCC cells, but an oncogenic role in BAF180-expressing ccRCC cells.

### BAF180's tumor-suppressive or -promoting activity depends on expression of HIF1α or HIF2α protein, respectively

**Figure 4:**
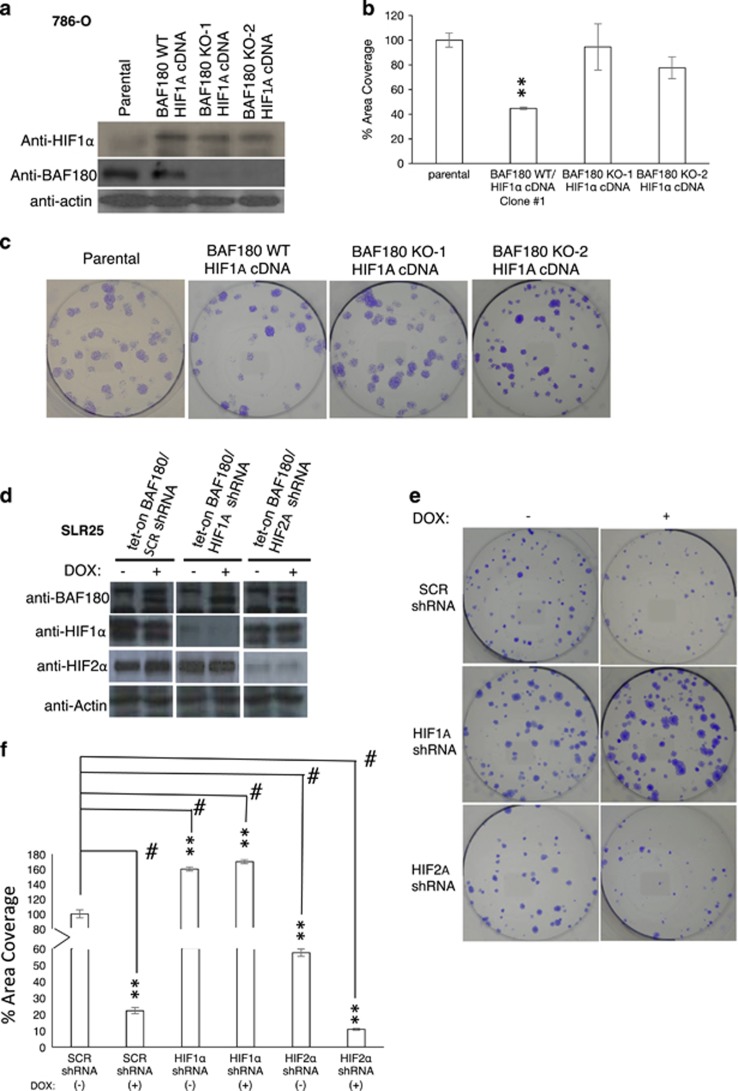
BAF180's tumor-suppressive or -promoting activity in ccRCC cell lines is HIF1α or HIF2α dependent. (**a**) Western blot analysis of HIF1α and BAF180 protein in parental 786-0 (parental), 786-0 with HIF1α re-expression (BAF180 WT/HIF1A cDNA) and 786-0 with HIF1α re-expression, but PBRM1 knockout by sgRNA #1, clone #1 and clone #2 (BAF180 KO-1 or -2/HIF1A cDNA). (**b**, **c**) Quantification (**b**) and photos (**c**) of clonogenic survival assay in the indicated 786-O cells. (**d**) Western blot analysis of BAF180, HIF1α and HIF2α proteins in SLR25/Tet-on BAF180 cells stably targeted with control, *HIF1A* or *HIF2A* shRNA in the absence of (−) or presence of doxycycline (+). (**e**, **f**) Quantification (**e**) and photos (**f**) of clonogenic survival assay of the SLR25/Tet-on BAF180 cells stably targeted with control, *HIF1A* or *HIF2A* shRNA in the absence of (−) or presence of doxycycline (+). All the samples are normalized to SCR non-targeting shRNA without doxycycline treatment.

To determine whether the loss of tumor-suppressive activity of BAF180 in H2 ccRCC cell lines is due to the loss of *HIF1A* gene expression, the H2 ccRCC cell line, 786-O, which normally expresses full-length BAF180, but lacks HIF1α expression, was stably transfected with a mouse *HIF1A* complementary DNA (cDNA) expression vector (BAF180 WT/HIF1A cDNA; Figure 4a). HIF1α re-expression reduced 786-O cell proliferation/survival compared with parental 786-O cells (Figures 4b and c), consistent with the reported tumor-suppressive role of HIF1α in ccRCC cell lines.^24, 25^ Next, *BAF180* was knocked out via CRISPR/Cas9 system to generate a cell line, BAF180 KO/HIF1A cDNA (Figure 4a). BAF180 KO/HIF1A cDNA 786-O cells exhibited increased cell survival/proliferation, compared with BAF180 WT/HIF1A cDNA 786-O cells (Figures 4b and c), demonstrating that loss of the tumor-suppressive activity of BAF180 in H2 ccRCC cells is due to the loss of *HIF1A* gene expression.

H1H2 ccRCC cell lines (RCC4 and SLR25) express both HIF1α and HIF2α, but lack BAF180 protein expression, and re-expression of BAF180 in these cells decreased cell survival/proliferation (Figure 2). To test if BAF180 tumor-suppressive function in H1H2 ccRCC cell lines depends on *HIF1A* expression, we generated SLR25/Tet-on BAF180/HIF1A KD cells; to test if BAF180's tumor-promoting activity is associated with *HIF2A* expression, we also generated SLR25/Tet-on BAF180/HIF2A KD cells, along with SLR25/Tet-on BAF180/SCR shRNA cells as a control (Figure 4d). In the absence of BAF180 expression (no doxycycline), *HIF1A* KD increased cell survival/proliferation compared with SLR25/SCR shRNA cells. In contrast, *HIF2A* KD decreased clonogenic survival/proliferation. BAF180 re-expression reduced SLR25/SCR shRNA cell survival/proliferation, which is consistent with data in Figure 2e. Expression of BAF180 in SLR25/HIF2A shRNA cells also reduced cell survival/proliferation compared with the same cells without BAF180 expression, suggesting that BAF180 protein works with the remaining HIF1α to further reduce cell survival (Figures 4e and f). Furthermore, expression of BAF180 in SLR25/HIF1A shRNA slightly increased clonogenic survival (*P*=0.0103) compared with the same cells without BAF180 expression, indicating that BAF180 and the remaining HIF2α protein cooperate, at least partially, to increase cell survival (Figures 4e and f). Thus, these data revealed that the function of BAF180 in ccRCC cell lines is dependent on the status of HIF1α or HIF2α expression.

### BAF180 inhibition reduces HIF1 and HIF2 target gene expression in Hep3B and ccRCC cells

**Figure 5:**
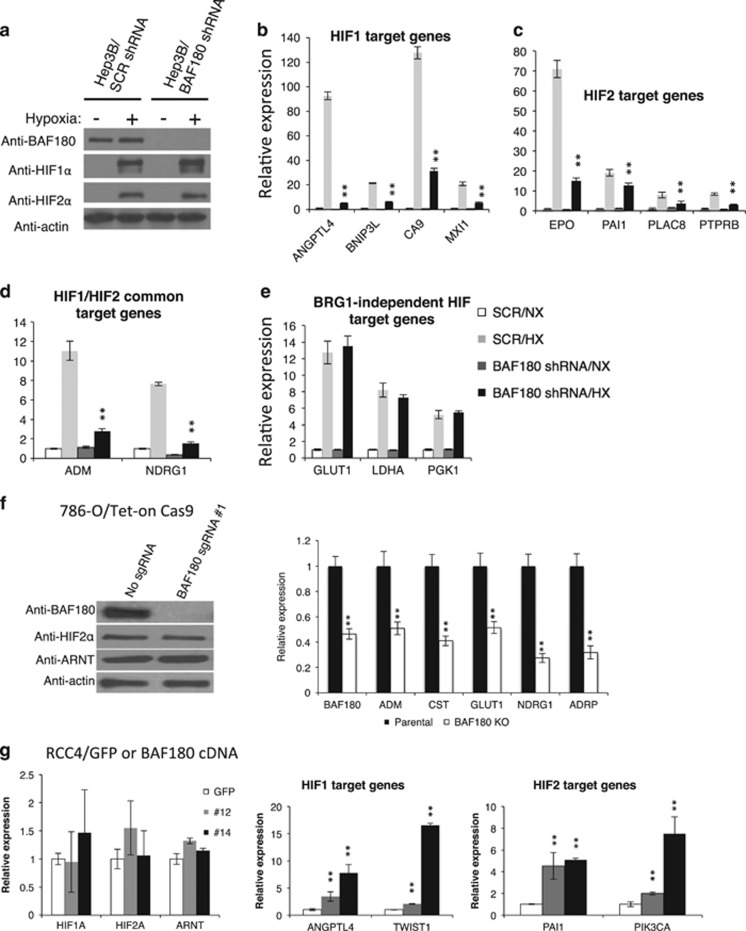
BAF180 is required for strong expression of HIF1 and HIF2 target genes in hypoxic (HX) Hep3B cells and pVHL-deficient ccRCC cell lines. (**a**) Western blot analysis of BAF180, HIF1α and HIF2α proteins in normoxic (NX) and HX Hep3B cells stably transduced with non-targeting control (SCR shRNA) or BAF180 shRNA. (**b**–**e**) qRT–PCR analysis of messenger RNA (mRNA) levels of HIF1 target genes (**b**), HIF2 target genes (**c**), HIF1/HIF2 common target genes (**d**) and BRG1-independent HIF target genes (**e**) in NX and HX Hep3B cells stably targeted with control or BAF180 shRNA. (**f**) qRT–PCR analysis of mRNA levels of known HIF2 target genes in 786-O/Tet-on Cas9 cells with no sgRNA or BAF180 sgRNA #1 (right). Expression levels of BAF180, HIF2α and ARNT protein in these cells were also shown (Left). (**g**) qRT–PCR analysis of mRNA levels of known HIF1 and HIF2 target genes, as well as HIF and ARNT in RCC4 cells stably expressing GFP (control) or BAF180 protein. Clones #12 and #14 express low and high BAF180 protein levels, respectively.

The ATPase, BRG1, is absolutely required for the function of the PBAF SWI/SNF chromatin remodeling complex; however, the accessory subunits such as BAF180 in the SWI/SNF complex, may or may not be important for a particular process.^30, 31, 32^ Although we have reported that the BRG1 knockdown significantly reduces HIF1 and HIF2 to activate their target genes,^23^ the role of BAF180 in HIF1 and HIF2 target gene activation remains unclear. To address this, we used Hep3B cells as a model, as they have a high level of HIF1α and HIF2α expression, and characterization of HIF target gene induction has been well established.^16, 33, 34, 35^ BAF180 shRNA significantly reduced BAF180 protein levels in normoxic and HX Hep3B/BAF180 shRNA cells (Figure 5a) and BAF180 knockdown markedly reduced the HX induction of HIF1 target genes (Figure 5b), HIF2 target genes (Figure 5c) and HIF1/HIF2 common target genes (Figure 5d), albeit the levels of reduction were gene specific. We have published that there is a subset of HIF target genes such as *GLUT1*, *LDHA* and *PGK1*, whose HX induction are not affected by BRG1 knockdown.^23^ Consistent with this published study, BAF180 knockdown did not alter the HX induction of these genes (Figure 5e).

Knockout of BAF180 in 786-O cells reduced the expression levels of HIF2 target genes (*ADM*, *CST*, *GLUT1*, *NDRG1* and *ADRP*; Figure 5f). Also KC-12/BAF180 shRNA and 769-P/BAF180 shRNA cells exhibited reduced HIF2 target gene expression in comparison with control shRNA (not shown). Conversely, BAF180 re-expression in RCC4 cells increased HIF1 and HIF2 target gene expression in a does-dependent manner (Figure 5g). Thus, BAF180 is important for HIF target gene activation in Hep3B, and in both H2 and H1H2 ccRCC cell lines.

### BAF180 contributes to chromatin remodeling of the CA9 promoter in HX Hep3B cells

**Figure 6:**
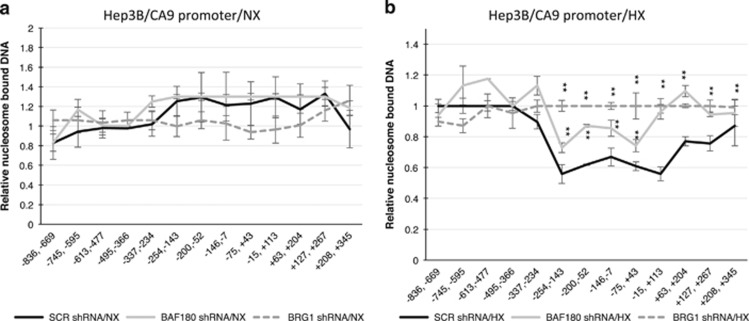
BAF180 expression is important for efficient de-association of nucleosome on the CA9 promoter in hypoxic (HX) Hep3B cells. (**a**, **b**) Nucleosome scanning assay of the CA9 promoter in Hep3B cells stably transduced with non-targeting SCR, or BRG1, or BAF180 shRNA under normoxia (**a**) or hypoxia (**b**). The location of the primer sets are shown relative to the transcription start site (+1). A region spanning −713 to −477 was used as an internal DNA-loading control, as the readouts in this region did not differ between normoxic (NX) and HX, nor between cells with WT BRG1 and BRG1 knockdown. Relative fractions of nucleosome DNA indicate the levels of nucleosome-bound DNA relative to those for an internal control.

We previously have shown that the PBAF SWI/SNF complex activates transcription of HIF target genes, including CA9 by decreasing nucleosomes associated with the HIF target gene promoter of the CA9 gene.^23^ To test the role of BAF180 in chromatin remodeling of HIF target genes, we performed a qPCR-based nucleosome scanning assays of the CA9 promoter. In Hep3B/SCR shRNA cells, hypoxia treatment significantly reduced nucleosome association on the CA9 promoter, especially in the region from −254 to +113, which is important for RNA polymerase II and HIF/ARNT binding (the HIF binding site is at −17; Figure 6b). HX Hep3B/BRG1 shRNA cells exhibited significant nucleosome association on the CA9 promoter, compared with HX Hep3B/SCR shRNA cells. Hep3B/BAF180 shRNA cells exhibited intermediate levels of nucleosome association on the CA9 promoter, compared with nucleosome association in HX Hep3B/SCR shRNA and Hep3B/BRG1 shRNA cells (Figure 6b). Thus, our findings reveal that BAF180 contributes significantly to chromatin remodeling of the CA9 promoter in HX Hep3B cells.

## Discussion

HIF1 displays tumor-repressive activity in established ccRCC tumors. Recently, it has been shown that a subset of ccRCC tumors still retain *HIF1A* gene expression;^24, 36^ however, it is unclear how these HIF1α-expressing ccRCC tumors tolerate HIF1 tumor-suppressive activity. Likewise, the *BAF180* gene is mutated in 40% ccRCC tumors, but the reason for *BAF180* mutations in these BAF180-deficient ccRCC tumors is not known. Furthermore, a subset of ccRCC tumors retain *BAF180* gene expression, the function of BAF180 in these BAF180-expressing ccRCC cells is also unknown. In this report, we determined that *BAF180* gene mutation, and the subsequent lack of BAF180 protein expression, is observed only in ccRCC cell lines that maintain full-length HIF1α protein expression. Re-expression of the BAF180 protein in these BAF180-deficient H1H2 ccRCC cells increases HIF1 target gene expression and inhibits cell proliferation/survival. Thus, our data indicate that mutation of *BAF180* is an alternative strategy for ccRCCs to reduce the tumor-suppressive activity of HIF1. However, BAF180 is expressed in H2 ccRCC cell lines in which the *HIF1A* gene is mutated. Reduction or knockout of BAF180 gene in these BAF180-expressing ccRCC cells reduces HIF2 target gene expression and cell proliferation/survival, indicating that BAF180 has oncogenic activity in this setting. Thus, our data indicate that *BAF180* is not a bona fide tumor-suppressor or oncogene, and BAF180's function in ccRCC is context dependent. These findings are very novel, as the current hypothesis accepted by most investigators is that BAF180 is a bona fide tumor suppressor and loss of BAF180 promotes cancer by re-targeting the SWI/SNF complex to a set of cancer-promoting genes that are not normally regulated by the SWI/SNF complex.^37^

Mutations in the *BAF180* gene or downregulation of *BAF180* gene expression has been observed in various cancer types, including ccRCC, suggesting a tumor-suppressor function.^7, 10, 11, 38, 39, 40, 41, 42^ Our data confirmed that *BAF180* is mutated in a subset of ccRCC cell lines (Figure 1a and Supplementary Figure 2a) and primary tumors (Figure 1b and not shown). Re-expression of *PBRM1* in BAF180-deficient ccRCC cell lines, both in stable or inducible manner (Figure 2), reduce ccRCC proliferation/survival, further confirming that BAF180 is a tumor-suppressive protein in the BAF180-deficient ccRCC cell lines, which is consistent with published papers.^7, 28, 29^

Although the previous studies^7, 28, 29^ did not address the molecular mechanism for BAF180 mutation in ccRCC, we have shown here for the first time that BAF180 mutation is to reduce HIF1's tumor suppressive in ccRCC. This conclusion is first supported by our novel observation that *BAF180* and *HIF1A* gene mutations are mutually exclusive in ccRCC cell lines (Figure 1a and Supplementary Figure 2a). We consider ccRCC cell lines expressing truncated HIF1α proteins as a HIF1α-deficient cell line, as a previous report indicates that the truncated HIF1α protein is not functional.^26^ By searching the literature, we have found additional ccRCC cell lines that exhibit mutually exclusive expression of BAF180 and HIF1α proteins, although these publications were focusing on BAF180 expression and made no connection between *HIF1A* mutation and *BAF180* mutation.^8, 43^ For example, H2 ccRCCs (SKRC-17, SKRC-59 and UMRC-6) express BAF180 protein, whereas H1H2 ccRCCs cell lines (CAKI-2 and UMRC-2) lack BAF180 expression.^43^ Further, H1H2 ccRCC cell lines RCC ER, RCC FG2 and RCC MF contain *BAF180* gene mutations.^8^ We also found two outliers (Supplementary Figure 2). SLR24 cells are double negative, whereas SLR22 cells are double positive, although both HIF1α and BAF180 protein are significantly reduced in SLR22 cells. We speculate that these outliers are formed in cells with initial BAF180 reduction (SLR22) or mutation (SLR24), followed by further *HIF1A* reduction (SLR22) or *HIF1A* mutation (SLR24).

Our experimental data (Figure 1a and Supplementary Figure 2) and our literature analysis above have established the mutually exclusive relationship of BAF180 and HIF1α protein expression in the majority (19/21=90%) of ccRCC cell lines, our own data (Figure 1b) and analysis of the published data sets (see below) also support such relationship in a subset of the primary ccRCC tumors. For example, by analysis of *HIF1A* copy number and exome sequencing,^10^ TCGA found that four ccRCC tumors are HIF1 activity deficient, due to loss of one *HIF1A* allele and have nonsense mutations in the second allele of *HIF1A* gene. Interestingly, we found that these *HIF1A* mutant ccRCC tumors express WT *BAF180* gene.^10^ Also Sato *et al.*^11^ reported 17 HIF1α-negative ccRCC tumors and we found that 13 out of the 17 (76%) HIF1α-negative ccRCC tumors express BAF180 proteins (one-tailed Fisher's exact test: *P*=0.0245). Consistent with these data, our own immunohistochemistry staining of BAF180 and HIF1α in ccRCC tumor tissues (BC0714a, US BioMax) found 7 HIF1α ^−^/BAF180^+^ and 22 HIF1α^+^/BAF180^−^ tumors out of a total of 66 ccRCC tumors.

The conclusion that BAF180 protein's tumor-suppressive activity in ccRCC is HIF1 dependent is also supported by functional studies, in which expression of HIF1α in H2 786-O cells switches BAF180 from a oncogenic protein to tumor-suppressive protein (Figures 4b and c), although knockdown of HIF1α in H1H2 SLR25 cells turns BAF180 from a tumor-suppressive protein to a tumor-promoting protein (Figures 4e and f).

Our data strongly support a notion that BAF180's activity in ccRCC is closely linked with the TFs HIF1 and HIF2. However, this conclusion does not exclude the possibility that BAF180-containing SWI/SNF chromatin remodeling complex could also potentially work with other TFs and regulate other genes/pathways under different conditions. In fact, multiple publications reported that BAF180 is required for p53 to activate p21 expression under condition of p53 activation.^29, 44, 45^ Although p53 can be activated in ccRCC,^29^ it is unlikely that p53 is activated in ccRCC cells under our study condition that cells are cultured under normoxia. In contrast, HIF is constitutively active in ccRCC cell lines under normoxia (Figure 1a). This might explain why the function of BAF180 in ccRCC is closely associated with HIF, not or less with other TFs such as p53.

In conclusion, we show, for the first time, that *HIF1A* and *BAF180* protein expression are mutually exclusive in ccRCC cell lines and some primary tumors. Further, our results indicate that BAF180's function in ccRCC is closely associated with HIF1 or HIF2 activity, the dominant TFs/pathway in the ccRCC tumors. These findings are of significance for personalized medicine, in which the inhibition of the BAF180 activity may be beneficial for H2 ccRCC patients, but ineffective for H1H2 ccRCC patients.

## Materials and methods

### Cell culture

HK2 cells were cultured in Hyclone (Logan, UT, USA) Dulbecco's modified Eagle's Medium (DME) and Ham's F-12 nutrient mixture 1:1 (1 × ) supplemented with 10% fetal bovine serum (FBS), 2.5 mM l-glutamine, 15 mM HEPES buffer and 1 × insulin–transferrin–selenium (ITS). 786-O, A498, RCC10, RCC4 and A704 cell lines were cultured in DMEM (Mediatech, Inc., VA, USA) supplemented with 10% FBS and other supplements. SLR21, SLR22, SLR23, SLR24, SLR25, SLR26, KC-12 and 769-P cell lines^46^ were cultured in RPMI1640 supplemented with 10% FBS and other supplements. Hep3B cells were cultured in MEM/EBSS (Thermo Scientific, Waltham, MA, USA) with 10% FBS and other supplements. When hypoxia treatment was needed, HEPES was added to the growth medium at final concentration of 25 mM and cells were placed in normoxia (Nx, 21% O_2_) or hypoxia (Hx, 1.5% O_2_) for 12–16 h for HIF target gene studies. RCC4, 786-O and SLR25 cells were authenticated by DNA profiling or ‘fingerprinting' by the University of Colorado DNA Sequencing and Analysis Core and have been determined to be mycoplasma free.

### Plasmid constructs and stable transfection

*BAF180* cDNA amplified from Hep3B cells was inserted into the pLEX-MCS (Thermo Scientific) or pCW57.1 vector (Addgene #41393, Cambridge, MA, USA; deposited by David Root laboratory). These lentiviral vectors were then transfected into 293T cells with other packaging plasmids to generate viruses that were used to obtain RCC4 or SLR25 cells expressing BAF180. The pcDNA3.1-hygro mouse HIF1A-Flag cDNA vector has been previously described.^16^ The vector linealized by FspI was transfected into 786-O cells by Lipofectamine (Invitrogen, Waltham, MA, USA). The stably transfected cells were generated by hygromycin B selection.

### shRNA-mediated knockdown of endogenous messenger RNAs

pLKO.1 lentivirus expressing the non-targeting scrambled shRNA (SHC202), *HIF1A* shRNA (TRCN0000003810), *HIF2A* shRNA (TRCN0000003803) or *PBRM1/BAF180* shRNAs (TRCN0000235892 or TRCN0000235890) were from Open Biosystems (Lafayette, CO, USA). Lentiviruses expressing shRNA were generated by transfection of the pLKO.1 shRNA construct with other viral protein expression vectors into 293T cells using TransIT (Mirus, Madison, WI, USA). The knockdown cells were generated by infection with the shRNA virus and puromycin selection.

### CRISPR–Cas9-mediated editing of *BAF180* gene in 786-O cells

pCW-Cas9 (doxycycline-inducible Cas9 expression; #5066) and pLX-sgRNA (lentiviral vector express AAVS1 sgRNA, #50662) were purchased from Addgene.^47^ pLX-sgRNA was first modified by PCR mutagenesis by replacing the AAVS1 sgRNA with two *Bsp*MI recognition sites to generate a sgRNA cloning vector, pLX-sgRNA-BspMI. Oligos for the PBRM1/BAF180 target sequence were: sgRNA#1 (5′-CACCGCTGACACTGCTGGAAGG-3′, 5′-AAACCCCCTTCCAGCAGTGTCAG-3′) and sgRNA#2 (5′-CACCGTCATTAGGGCACCAAAG-3′, 5′-AAACTCGCTTTGGTGCCCTAATG-3′). Each pair of target oligos was first annealed and then ligated into pLX-sgRNA-BspMI vector that had been cut with *Bsp*MI. To generate 786 cells with *BAF180* knockout, first, 786-O/Tet-on Cas9 cells were generated by transducing 786-O cells with a pCW-Cas9 lentivirus. Subsequently, 786-O/Tet-on Cas9 cells were transduced with *BAF180* sgRNA lentivirus and selected for 7 days with blasticidin S HCl (12 μg/ml) and 1 μg/ml doxycycline. Individual cells were then plated in 96-well plates. BAF180 expression in each subsequent clone was determined by western blotting, from which ~5% clones were determined to be BAF180 negative. Clones with loss of BAF180 protein expression were further confirmed by sequencing of the *BAF180* genomic locus.

### Western blotting analysis

Protein lysates were prepared with whole cell extraction buffer (150 mM NaCl, 5 mM EDTA, 50 mM Tris pH 8.0, 0.1% SDS, 1 mM PMSF and 1 × Pierce protease inhibitor cocktail). Total protein concentration was determined using bicinchoninic acid assay reagents (Pierce, Waltham, MA, USA). For western blot analysis, 25 μg of total protein was separated on an 8% SDS–polyacrylamide gel electrophoresis gel. The following primary antibodies were used: anti-HIF1α polyclonal (pAb) (NB100-134; Novus Biologicals, Littleton, CO, USA), anti-BAF180 pAb (NB100-79833; Novus Biologicals), anti-HIF2α mAb (D9E3, Cell Signaling Technology Inc., Danvers, MA, USA), anti-ARNT Ab (NB100-124, Novus Biologicals) and anti-actin pAb (SC-1616; Santa Cruz, Santa Cruz, CA, USA).

### RNA isolation and reverse transcriptase–quantitative PCR analysis

RNA was extracted from cells using the RNeasy Plus mini kit (Qiagen, Venlo, Netherlands) followed by reverse transcription using iScript Advanced cDNA Synthesis Kit (Bio-Rad, Hercules, CA, USA). Quantification of messenger RNA levels was performed by reverse transcription quantitative PCR (RT–qPCR) using iQ Universal SYBR Green Supermix (Bio-Rad) and CFX384 Real Time System (Bio-rad). All primer sets for RT–qPCR were validated for their specificity and amplification efficiency (85% to 110%) using melt curve analysis, RT–qPCR product sequencing and standard dilution analysis (Supplementary Table 1). The qPCR results were analyzed using the ΔΔCT method using 18S ribosomal RNA and beta actin messenger RNA as reference genes and presented in relative to samples from control cells. At least three independent experiments were performed for all the results presented in this paper.

### Detection of *HIF1A* gene deletions in ccRCC cell lines

Genomic DNA was isolated from each ccRCC cell line using a QIAamp DNA mini kit (Qiagen). Twenty ng of genomic DNA was used in each qPCR reaction to quantify individual *HIF1A* exons using primer stets (Supplementary Table I) located in exons 2–15. Results of *USF1* exon 11 and *LMNA* exon 7–intron 7 junctions were used as genomic DNA concentration controls. The DNA from HK2 cells was used as normal DNA content calibrator.

### Clonogenic survival assay

Cells (250) were plated per well of a six-well plate, and cultured in 1% FBS and 1 × insulin–transferrin–selenium for 7–10 days. For Tet-on BAF180-expressing cells, PBS or 1 μg/ml doxycycline were added to the medium. After 7–10 days, cells were washed with PBS once and stained with crystal violet (50% glutaraldehyde and 0.5% (w/v) crystal violet). Quantification was performed using MetaMorph software (Nashville, TN, USA) to measure the total area covered by colonies.

### Immunohistochemistry analysis of BAF180 and HIF1α in ccRCC tumors

ccRCC Tissue Microarrays were obtained from US Biomax, Inc (cat. #BC07014a). Immunohistochemical staining using HIF1α (Thermo Fisher Scientific, Waltham, MA, USA, H1alpha67) or BAF180 (Novus Biologicals; NB100-79833) antibodies was performed on two slides containing the same tumors according to standard immunohistochemistry protocols.

### Nucleosome scanning assay

Hep3B cells expressing shRNA against *BAF180* or *BRG1*, or non-targeting shRNA (~60% confluency) were cultured under normoxic or HX conditions for 16 hours and nucleosomal DNA was isolated using the EZ Nucleosomal DNA prep kit (Zymo Research, Irvine, CA, USA). The primer sets (Supplementary Table 2) and the procedure for quantification of the CA9 promoter region were described previously.^23^

### Statistical analysis

One-way analysis of variance followed by *post hoc* test (two-sample *t*-test with Bonferroni correction) was performed for statistical analysis unless otherwise stated. Error bars in figures indicate s.d. Asterisks indicate statistical significance as follows: **P<0.01, *P<0.03. Controls for statistical analysis are specified in each figure. All experiments were performed at least three separate times.
