# Supplementary material for: Context-dependent role for chromatin remodeling component PBRM1/BAF180 in clear cell renal cell carcinoma
Source: Oncogenesis. 2017 Jan 16;6(1):e287–. doi: 10.1038/oncsis.2016.89 (PMC5294252; doi:10.1038/oncsis.2016.89)
Supplement: Supplementary Figure and Table legends [file oncsis201689x1.docx]

**Supplemental Fig.1**. qPCR analysis of levels of individual exons of *HIF1A* gene in genomic DNAs isolated from indicated ccRCC cell lines. Relative DNA contents were normalized to *LMNA* and *USF1* genes as these genes were not amplified or deleted in ccRCC cells. In addition, the DNA content of the *HIF1A* exons from HK2 cells were used as calibrators as HK2 cells are normal kidney epithelial cells. Exons with a relative DNA content at 1, 0.5 or close to zero indicate normal, loss of one copy, or loss of both alleles of *HIF1A* gene in ccRCC cell lines.

**Supplemental Fig. 2**. Western blot analysis of BAF180 and HIF1α proteins in indicated ccRCC cell lines. Cells express HIF2α, but not functional/full-length HIF1α are labeled as H2, cells express HIF2α and full-length HIF1α are labeled as H1H2. SLR24 lost both HIF1α and BAF180 protein expression while SLR22 exhibited significant reduction of both BAF180 and HIF1α protein. Normoxic and hypoxic Hep3B cells are controls for BAF180, HIF1α, and HIF2α detection.

**Supplemental Table I**. qPCR primers used in the study.

**Supplemental Table II**. qPCR primers used for NUSA assays.
