# Supplementary figures and images for: Context-dependent role for chromatin remodeling component PBRM1/BAF180 in clear cell renal cell carcinoma
Source: Oncogenesis. 2017 Jan 16;6(1):e287–. doi: 10.1038/oncsis.2016.89 (PMC5294252; doi:10.1038/oncsis.2016.89)

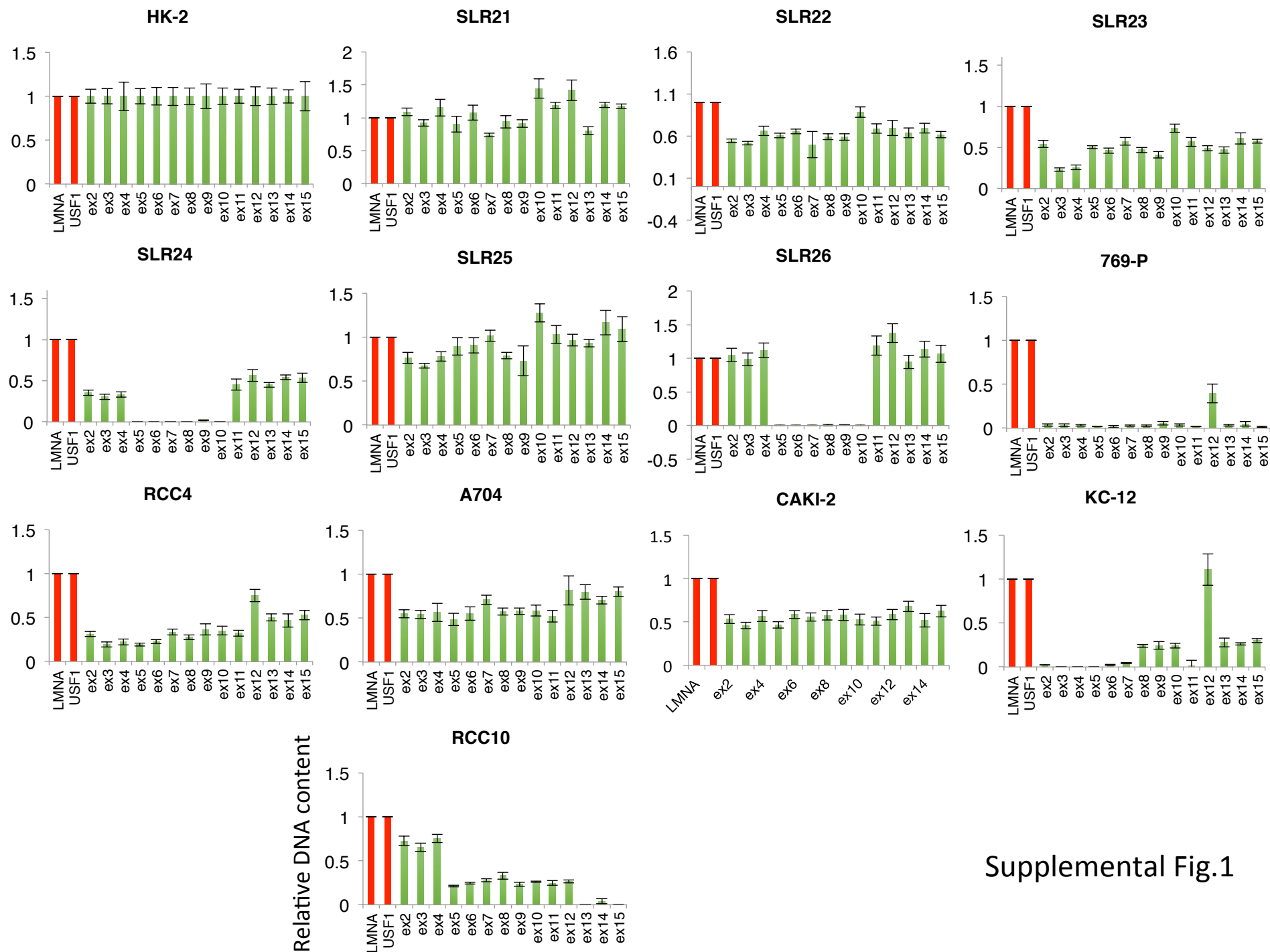

Supplemental Fig.1

Supplement: Supplementary Figure 1 [file oncsis201689x2.pdf]

Supplemental Fig.2

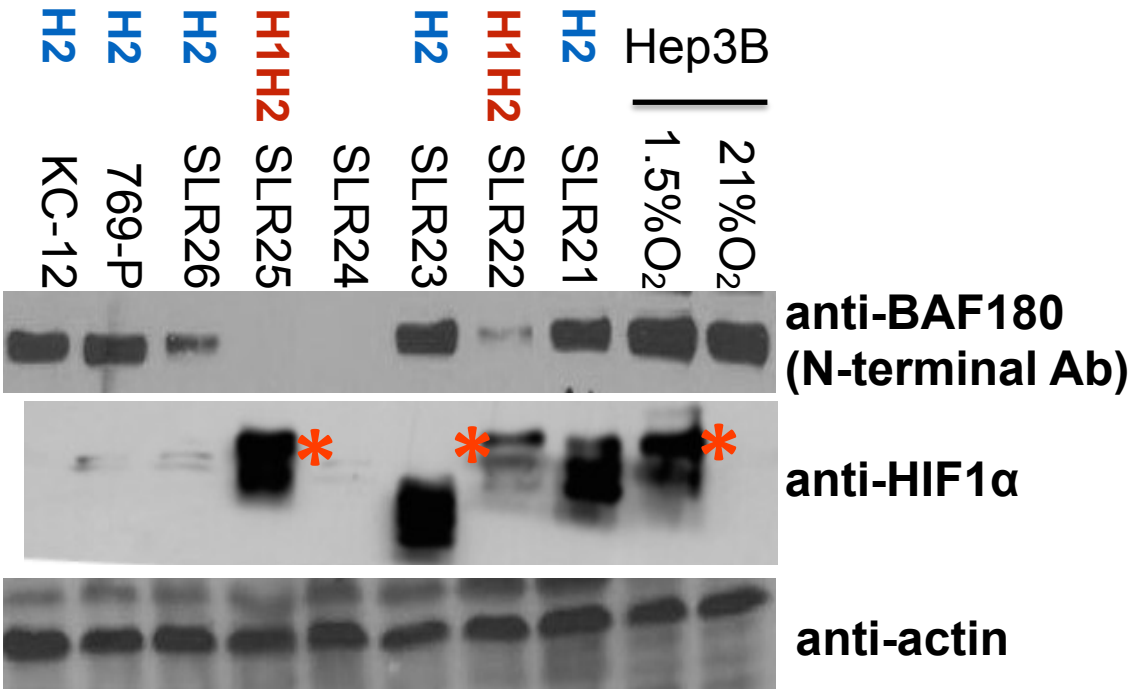

Supplement: Supplementary Figure 2 [file oncsis201689x3.pdf]
